# On the Capability of Smartphones to Perform as Communication Gateways in Medical Wireless Personal Area Networks

**DOI:** 10.3390/s140100575

**Published:** 2014-01-02

**Authors:** María José Morón, Rafael Luque, Eduardo Casilari

**Affiliations:** Departamento Tecnología Electrónica, University of Málaga, ETSI Telecomunicación, 29071 Málaga, Spain; E-Mails: rluque@uma.es (R.L.); ecasilari@uma.es (E.C.)

**Keywords:** wireless personal area networks, Bluetooth, SPP, HDP, smartphone, Android, performance evaluation

## Abstract

This paper evaluates and characterizes the technical performance of medical wireless personal area networks (WPANs) that are based on smartphones. For this purpose, a prototype of a health telemonitoring system is presented. The prototype incorporates a commercial Android smartphone, which acts as a relay point, or “gateway”, between a set of wireless medical sensors and a data server. Additionally, the paper investigates if the conventional capabilities of current commercial smartphones can be affected by their use as gateways or “Holters” in health monitoring applications. Specifically, the profiling has focused on the CPU and power consumption of the mobile devices. These metrics have been measured under several test conditions modifying the smartphone model, the type of sensors connected to the WPAN, the employed Bluetooth profile (SPP (serial port profile) or HDP (health device profile)), the use of other peripherals, such as a GPS receiver, the impact of the use of the Wi-Fi interface or the employed method to encode and forward the data that are collected from the sensors.

## 1. Introduction

mHealth (mobile health) systems are emerging as an alternative to conventional medical monitoring applications, such as Holter monitors, which have traditionally been used to collect off-line biosignal data. mHealth systems are normally founded on the utilization of wireless sensors and a personal device (presently, a smartphone or tablet), which is in charge of forwarding the data sent by the sensors to the monitoring point.

As explained in [1], mHealth facilitates the continuous real-time patient tracking and data processing of the biosignals as a part of the diagnostic procedures. Due to the popularity and decreasing costs of mobile communications, mHealth systems are a good candidate for optimizing the monitoring of patients with chronic diseases or in a rehabilitation phase (computer-assisted rehabilitation). Moreover, mHealth solutions also avoid some important limitations that hinder the acceptance of conventional systems for continuous biosignal monitoring (unshielded wires between the sensors and the processing unit, absence of a system that seamlessly integrates individual sensors from different vendors, radio interferences in the wireless channels shared by the devices, lack of support for massive collection of data and knowledge generation, *etc.*). Simultaneously, as standard communication protocols are employed and a specific device for collecting the sensors' data has not been designed, mHealth solutions appreciably reduce the developing time of the monitoring systems.

Thus, the development of mobile platforms for healthcare applications has focused the interest of many research projects during the last years [2]. Although every platform has its own characteristics, the vast majority responds to a common three-level architecture. The first level is formed by one or several wireless sensors. The sensors are organized in a wireless body area network (WBAN) or a wireless personal area network (WPAN) [3], which is coordinated by a personal device. Thus, this second-level device assumes the role of a coordinating “hub” or gateway, that gathers the biosignals from the sensors, preprocesses the received data and retransmits them (also in a wireless way) to the third-level point: a central server. This server, which is normally Internet-connected, enables the online remote tracking and diagnosis of the patients.

As noted in [4], the widespread use of mobile phones and tablets, which are getting more and more popular in daily life, has contributed to the fact that mHealth technology has become the most representative application of WBANs in the medical field in recent years. According to the authors of [4], the adoption of smartphones, which are characterized by the integration of all the necessary technologies in an mHealth system, avoids the development of specific additional devices, reducing system costs, while increasing the user comfort. Similarly, the authors of [3] agree with this line of argument, *i.e.*, the capabilities of present smartphones (powerful processors, large memory capacity and multiple network interfaces, such as Bluetooth, Wi-Fi and cellular communications, relatively small costs, *etc.*) have fostered the adoption of this type of device as the gateway between the sensors and the remote servers in many experimental architectures of WBANs and WPANs. In this regard, the authors in [1] describe the advantages of the use of smartphones for ubiquitous health monitoring. The authors remark that its application to this area represents a promising field with the potential to significantly change healthcare systems and make them more cost-effective. Regarding WBAN or WPAN technologies, the authors of [5] highlight their aptitude to bring about a radical change in the way of providing healthcare in several environments. As a sample of this potential, the authors mention a report by ON World Inc. that estimated that wireless sensor networks could reduce annual healthcare costs by US$25 billion in 2012. The same firm has recently forecast that 18.2 million health and wellness WSNs will be shipped worldwide in 2017, generating more than US$16 billion in annual revenue [6].

The authors in [3] summarize the main implications of the utilization of mobile phones in healthcare services. In spite of the emergence of mobile applications, the authors comment on different unresolved technical issues that may hamper the adoption of mobile technologies for health-related purposes. These issues include aspects, such as usability, connectivity, security policies, *etc.* As is remarked in [2], most research works and experiences dealing with this subject basically assume the proper functioning of smartphones as health monitors, although these devices have not been designed for this function.

In this sense, this article presents a prototype of a health telemonitoring system based on commercial Android smartphones that act as information gateways. The main goal of the article is to thoroughly evaluate and characterize the performance of this type of technology under different usage scenarios.

The selected technology for communication by the smartphone and the medical sensors is Bluetooth (BT). Together with cellular telephony (normally 3G) and Wi-Fi, Bluetooth is one of the three wireless standard interfaces integrated in the vast majority of commercial smartphones. Although it was not conceived of for sensor networks, Bluetooth is clearly a more energy-efficient technology than 3G and Wi-Fi. Other low consumption radio protocols (ZigBee, ANT, wirelessHART, 6lowPAN, R4FCE, *etc.*) are not widely employed yet and, most importantly, have not been incorporated as communication interfaces to existing smartphones until now. In this sense, Bluetooth is a compromise between consumption and available bandwidth for point-to-point short-range transmissions. In addition, Bluetooth is the dominating technology in the market of wireless medical sensors. There are already more than 40 million Bluetooth-enabled healthcare devices on the market from leading manufacturers, like 3M, A&D, Nonin and Omron [7]. Nowadays, the most straightforward, cost-effective and seamless way to create a medical personal area network based on a smartphone is by means of Bluetooth connections. The use of other wireless technologies obliges the developing of specific hardware modules and heavily reducing the usability of the smartphone by incorporating external wired radio interfaces.

This paper is organized as follows: Section II reviews the related literature on the architectures of WPAN or WBAN that utilize smartphones to monitor biosignals, especially those works that carry out a specific evaluation of the technical performance of the prototypes. Section III describes the structure and components of a system conceived of to monitor the signals from different Bluetooth sensors. Section IV details the metrics that were employed to characterize the performance and discusses the obtained results of the test phase. Finally, Section V draws the main conclusions of the paper, comments on some ongoing research topics and proposes some guidelines to be considered for the development of this type of telecare system.

## 2. Related Work

A wide variety of examples of the usage of smartphones to record or monitor parameters related to certain diseases can be found in the literature, such as the experiences described in [8–16].

The authors of [17] point out that health tracking and management is an area that could take advantage of smartphone-based technology. In that particular case, a smartphone is utilized for the physiological monitoring of breathing signals. In the paper, the authors describe an implementation of a physiological audio processing application using an Android smartphone that is connected to an analog electronic stethoscope.

The study in [8] presents a portable ECG (electrocardiography) monitor that sends the ECG waveform to an Android smartphone via Bluetooth (BT), by using the BT serial port profile (SPP). The smartphone is employed to display the ECG waveform and is responsible for storing it together with a timestamp and location information on a non-volatile SD (secure digital) memory card. Additionally, the ECG data are transferred to a cloud computing system (specifically, Apache Hadoop, an open-source software framework for distributed applications).

In [9], the authors also describe an ECG monitor based on a smartphone. However, in this case, the deployed architecture employs wireless bipolar body electrodes that require a Simplici-TI-Bluetooth converter to be integrated with a Windows Mobile smartphone.

Another paper [16] details the design of a monitoring system based on a WBAN that uses measurements from one lead ECG sensor to diagnose apnea episodes. An Android smartphone is employed to manage the Bluetooth connectivity of the ECG sensor, to process the received signal and to forward the ECG data to a server.

The work in [10] comments on a telecare system that performs the monitoring of patient vital signs and daily activity. The system consists of a wrist-worn device, which provides the heart rate and the blood oxygen level, an Android smartphone and a web-based information system. The communication between the monitor and the smartphone is again accomplished via Bluetooth.

The authors in [11] presents BEAT (Bio-Environmental Android Tracking), another telecare prototype that has been developed using Android. This system also integrates an ECG device that is connected to the smartphone by Bluetooth (through RFCOMM, radio frequency communications). The functionality supported by BEAT covers local storage, data analyses, real-time monitoring, feedback to the user and management of emergency responses. In their study, the authors include a basic evaluation of two technical performance parameters: the required storage space and power consumption.

As it relates to the performance evaluation of this type of mHealth application, the authors in [18] present a comparison of the different methods of data delivery provided by the Android platform, aiming at selecting the most suitable method to be used for the transmission of digital health screening forms. The data considered by the authors were obtained from an accelerometer sensor and a GPS.

Furthermore, in order to compare the four major mobile platforms (Symbian OS, Windows Mobile, Android and iPhone), the authors of [19] evaluate the resources demanded by an application to collect, visualize and monitor data from a body temperature sensor, in both real-time and off-line modes.

The authors of [2] highlight the fact that a smartphone, which is a key element in most of the proposed architectures for health monitoring, has other capabilities (apart from calls), such as music and video playback. The use of these applications may impact the role of the smartphone in healthcare applications. In order to evaluate the capability of smartphones to simultaneously support both the typical tasks of a smartphone and the monitoring processes, the paper investigates different performance metrics (such as battery consumption, data processing or packet loss rate) in different scenarios that simulate real life. As a result of the experiments, the authors infer that combining both functionalities is not always feasible for some applications. Consequently, they propose a specific architecture called PHM-Gate (Personal Health-Monitoring Gateway). This architecture introduces a specific device that interacts and handles all the information received from the sensors. Therefore, once the sensors' biosignals are processed, they are conveyed to a smartphone via Bluetooth. The smartphone, in turn, retransmits the signals to the servers that centralize the information in the system. However, the need for a specific hardware to act as a gateway between the smartphone and the sensors increments the developing time and the costs of the system, while it does not maximize the wide availability of resources that existing smartphones offer to the user.

This paper analyzes if the conventional functionalities of commercial smartphones are influenced when they are utilized as gateways or “Holters” in telecare applications. In particular, the main goal of this paper is the characterization of the technical performance of a wireless personal area network (WPAN). In contrast with other similar works ([11,18,19] or [2]), the profiling is focused on the CPU and power consumption, which are measured under different operational conditions.

## 3. System Description

**Figure 1.**
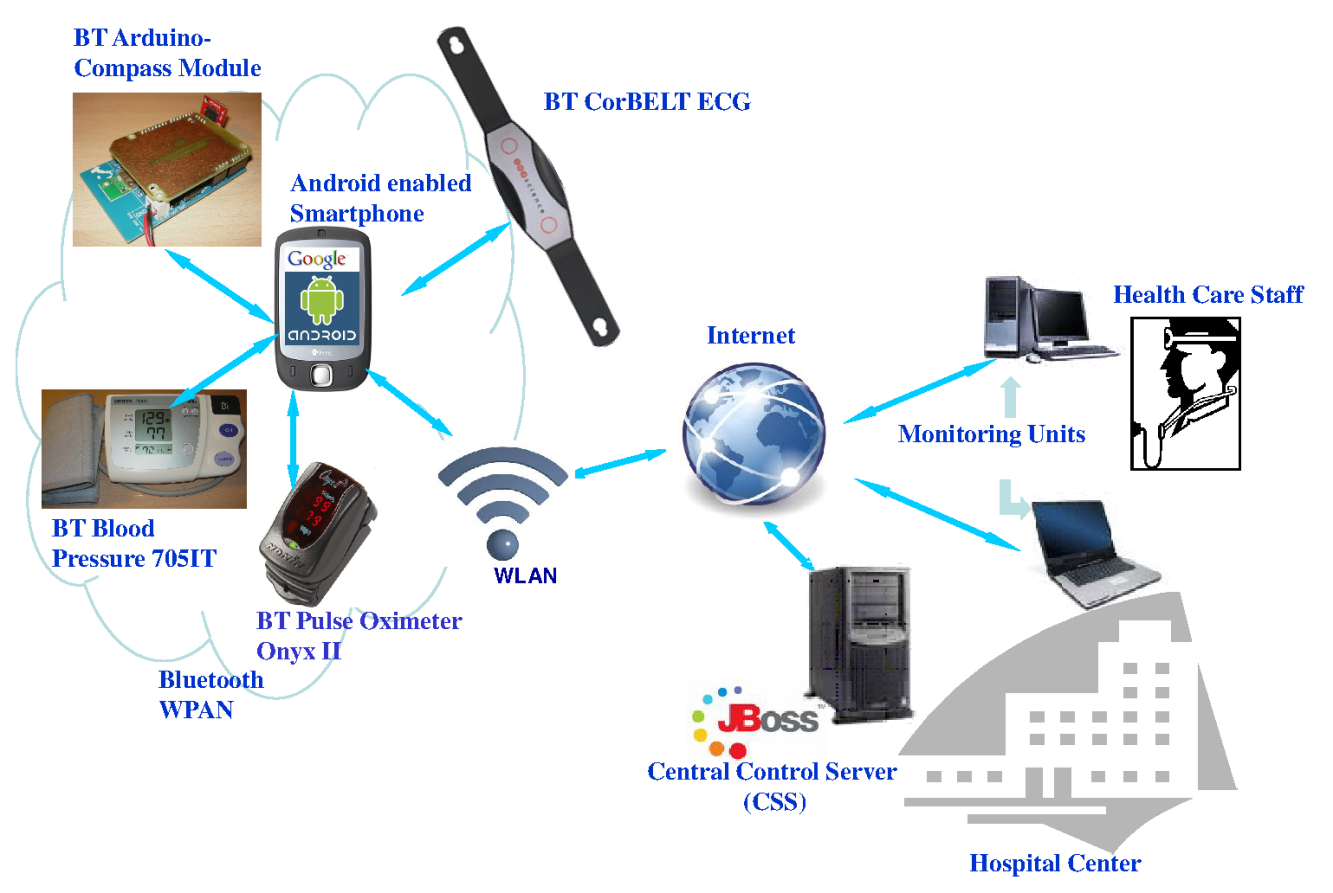
System architecture of the developed prototype.

The developed prototype, sketched in Figure 1, is based on a Bluetooth WPAN. The proposed architecture consists of three components: (i) a wireless personal area network that is transported by the user that is going to be monitored; (ii) a central control server (CCS); and (iii) a web application for remote control and monitoring. Figure 1 shows these components, which are described in the following subsections:

### 3.1. Wireless Personal Area Network (WPAN)

The WPAN is, in turn, integrated by (i) three Bluetooth-enabled sensors: a pulse-oximeter, a single derivation ECG (electrocardiography) sensor and a blood pulse monitor; (ii) an Arduino module with a three-axis compass and a Bluetooth interface; and (iii) a GPS-enabled smartphone, which will perform as the WPAN coordinator.

In order to increase the flexibility of this architecture, the simultaneous connection of the whole set of sensors is not mandatory. Thus, the system can work just with the smartphone and the pulse-oximeter. The following paragraphs comment on the basic characteristics of these elements in the WPAN:

#### 3.1.1. Bluetooth-Enabled Medical Sensors

##### Pulse-Oximeter

The employed Bluetooth pulse-oximetry sensor is the Onyx II 9560 model by Nonin [20]. In order to ensure the interoperability between devices from different manufacturers, the Bluetooth standards specify the so-called BT profiles. A BT profile defines the protocols and procedures that must be implemented to guarantee the data exchange under different typical application scenarios. This model of pulse-oximeter implements two different profiles, commonly utilized by BT communications in mHealth applications:

- SPP (serial port profile): SPP is by far the most utilized BT profile, and it is implemented in a great variety of commercial BT devices. SPP enables the connection between two Bluetooth-enabled nodes by emulating RS-232 serial ports through the RFCOMM protocol. In the case of this pulse-oximetry sensor, the devices utilize a specific algorithm, called SP (SmartPoint), to determine when a high quality measurement is ready to be transmitted. Among the different operating modes provided by the device operating under SPP, we have selected two: (i) a basic configuration—Mode 8, (M #8)—in which a four-byte packet (containing the heart rate, the oxygen saturation or SpO2 (Pulse Oximeter Oxygen Saturation) and some additional data, such as the battery status) is generated once per second; (ii) a “verbose” configuration—Mode 2, (M #2)—of continuous transmission for which five-byte packets are sent at a rate of 75 packets per second. Under this extended mode, extra data (such as the plethysmogram, the sensor status or control information) are also added to the packets (apart from the basic parameters conveyed in the basic mode).
- HDP (health device profile): This Bluetooth profile is compliant with the ISO/IEEE 11073-20601 Personal Health Data Exchange Protocol. In particular, the device follows the ISO/IEEE 11073-10404 normative, which specifies the communication between personal telehealth pulse oximeters and computer engines. When the HDP profile is selected, as in the basic configuration with SPP, the pulse-oximetry sensor transmits the data corresponding to the oxygen saturation and the heart rate.

It should be noted that the main difference between these two profiles basically lies in the fact that under the SPP profile, the information is encoded in a proprietary, manufacturer-specific format. Conversely, by using the HDP profile, the coding of medical data follows the ISO/IEEE 11073 group of standards.

##### ECG sensor

A Bluetooth single derivation electrocardiography sensor (CorBelt), manufactured by CorScience, has also been integrated in the prototype [21]. This device, which also supports Bluetooth SPP, samples the ECG at a 200 Hz frequency with a 12-bit resolution. ECG information, together with the measured heart rate, is encapsulated in 224-byte packets.

##### Blood pressure monitor

The employed model was the Bluetooth-enabled CorScience 705IT BT upper arm device. This model offers both SPP and dial-up Bluetooth profiles. SPP (operating as a slave) was selected for the application. Under this profile, the sensor normally remains in a standby mode waiting for a request for the value of the measured blood pressure.

#### 3.1.2. Arduino-Compass Module

**Figure 2.**
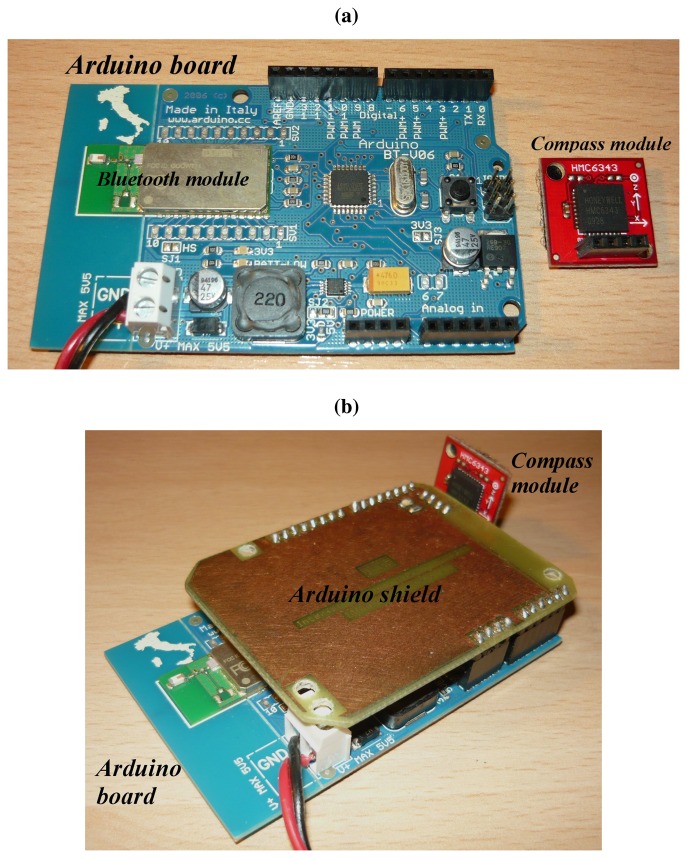
Employed Arduino-compass module. (**a**) BT (Bluetooth) Arduino module and Honeywell HMC6343 compass; (**b**) Assembled module.

The aim of this module, shown in Figure 2, is to provide the measurement of the absolute angular position and to send it via Bluetooth.

This module is composed by:

- An Arduino-BT [22] OSHW (open source hardware) developing board, based on the Atmel ATmega168 micro-controller and the Bluegiga WT-11 Bluetooth interface;
- A Honeywell HMC6343 [23] digital compass with I2C communication interface [24].

A 5 V DC-source was used to power the Arduino module (although a battery could have been alternatively employed).

#### 3.1.3. Android-Enabled Smartphone

A specific application has been developed in Android [25] for the smartphone that coordinates the WPAN. Android is an open-source software stack intended for touchscreen mobile devices (mainly smartphones and tablets) [26]. The Linux-based architecture of Android has allowed for extending all the potentialities and know-how of Linux to the mobile industry. Furthermore, Android applications are developed in a customized version of Java, one of the most popular general-purpose programming languages. As a consequence, since its release in 2007, the Android operating system (OS) has been widely accepted by hardware vendors, software developers and non-expert users. In fact, during the first-quarter of 2013, Android accounted for 75 percent of the mobile shipments [27]. This figure implies an increase of more than 15 percentage points in the market share with respect to the same quarter of the previous year, which illustrates the rapid expansion and the dominating position of this OS [27]. According to the global intelligence firm, IDC (International Data Corporation), Android will remain the leading software technology for smartphones at least through 2016.

**Figure 3.**
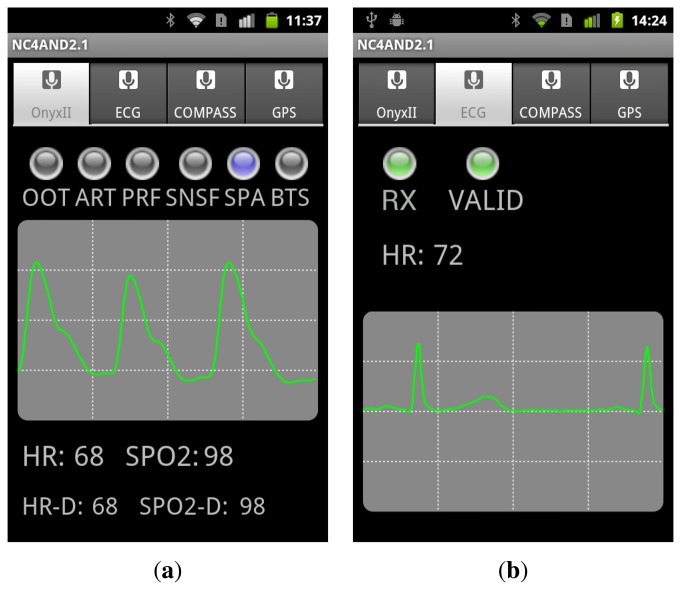
Snapshot of the interface in the smartphone showing the signals sent by the sensing devices that integrate the Bluetooth wireless personal area network (WPAN). (**a**) Pulse-oximeter; (**b**) electrocardiography (ECG) CorBELT.

The developed Android application offers two operation modes:

- Local mode: Under this configuration, the smartphone screen displays the fully detailed information received from the connected sensors. Figure 3 illustrates two examples of the views of the application when the data flowing from the pulse oximeter and the ECG are being represented.
- Remote mode: For this mode, the application in the smartphone performs as a gateway between the sensors and the server and offers a simplified view of the data on its screen. Thus, it retransmits to the central control server the data received from the sensors together with GPS position information. In this sense, in the case that the smartphone does not integrate a GPS device, the data about the user's location can be obtained from the cellular network or from an existing Wi-Fi access point, depending on availability. Similarly, the gateway acts as a relay point to redirect the configuration commands sent by the server to the sensors.

In this architecture, it is worth noting that the pulse-oximeter, the electrocardiograph and the Arduino-Compass module are utilized for continuous monitoring, while the blood pressure sensor is eventually employed for sporadic measurements, whose results are also transmitted to the server if a remote monitoring has been scheduled.

### 3.2. Central Control Server (CCS)

The central control server, deployed on JBoss 5.1.0 GA server [28], consists of a single Java servlet that interfaces between the WPAN transported by the patient and the web application. For communication with the application, we employed the JMS (Java message service) API (application programming interface) [29], namely, Queues and Topics. The CCS is responsible for the management of both elements for every monitored user. In particular, the CCS servlet is in charge of:

- Publishing the objects that encapsulate the tracking and monitoring information received from the patient in the topic corresponding to the patient. Thus, data are available for the web application as soon as they are required.
- Reading and retransmitting the commands stored by the web application. These commands, which regulate the sensors' activity, are inserted in the queue associated with the corresponding patient and, then, retransmitted to the WPAN. A command is encapsulated in the response to the HTTP (Hypertext Transfer Protocol) POST request received from the patient for whom it is targeted.

### 3.3. Remote Control and Monitoring Web Application

**Figure 4.**
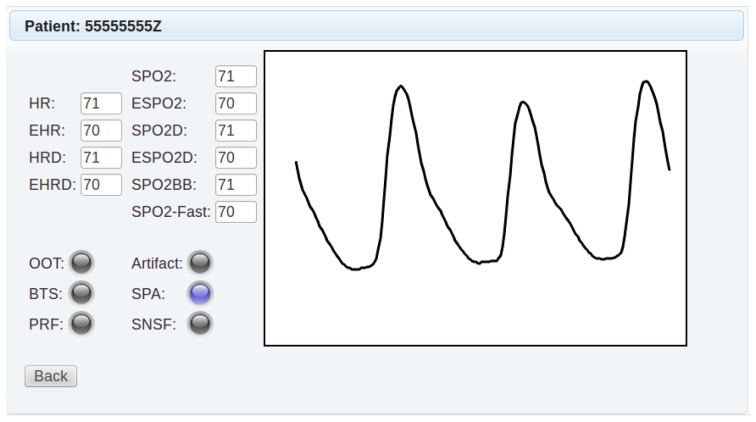
Screenshot of the interface showing the detailed pulse-oximetry data of a patient.

The system includes a web application for the remote control and monitoring of the sensors. The application subscribes to the JMS topics in order to obtain patient data, while it subscribes to JMS queues to send configuration commands to the sensors. The application was implemented with the JSF (JavaServer Faces) framework and enhanced with graphic components from the open source PrimeFaces project [30]. The information is given to the user through two different views:

- The basic monitored parameters of a selected set of patients are condensed in a summarized view.
- The application shows in a detailed view the location of the patient in Google Maps, as well as the data from the sensors. As an example, Figure 4 shows the information concerning the pulse-oximeter when it operates in the basic mode. In addition, the application enables the remote configuration of the data transmission period of the sensors, as well as other parameters, such as the operating mode of the pulse-oximeter or the numerical thresholds used for arrhythmia detection.

## 4. Evaluation Tests

As previously stated, the smartphone is proposed to be utilized for a completely different function from that for which it was conceived: a network coordinator node in a WPAN of medical sensors. In this section, we offer the results of a complete benchmarking of different commercial smartphones when they are employed for such purpose. The performed analyses have been divided into three categories:

1. Connectivity capacity (Section 4.1): For a selection of Android phones, we checked the actual capability of the devices to connect to the sensors via Bluetooth.
2. Benchmarking when the smartphone is employed as a health monitor (Section 4.2): This set of tests are oriented to characterize the resource consumption when the smartphone is acting as a health monitor.
3. Compatibility with standard functionality (Section 4.3): The goal of the tests encompassed in this group is to verify if the smartphone, when used as a monitoring unit, is able to develop other conventional functions (*i.e.*, play an audio file or make a call) properly.

The measuring method was as follows. Every experiment is based on the execution of the monitoring application of the smartphone under a particular configuration of the sensor network and the transmission mode (local, remote) of the system. The performance metrics are obtained by the Diagnosis-System Information [31] application provided by the Android operating system. This application samples the system state at regular time intervals (of about 5 s). This information is cached and averaged every 30 s (six samples). Thus, after an operation time of 150 min, we again average the obtained 300 samples of 30 s to compute the final performance metric. Experiments are repeated for three to 10 times, until the confidence interval (at 95%) of the measurement is under 5% of the final mean value of the obtained series.

In all the considered scenarios for the local and remote operational modes, the computational resources required by the application (expressed in terms of the percentage of use of the CPU, the utilized bandwidth and the expected duration of the battery) were measured. The battery lifetime is extrapolated from the measured battery state of charge during the experiment. In order to define a pessimistic estimator for the battery lifetime, we assume that the battery is exhausted when it is less than 5% charged. Aiming at validating this estimation, we have also run a benchmark test consisting of measuring the voltage supplied by the smartphone battery for a full discharge cycle. Results show that the supply voltage always remains over an acceptable value until that critical 5% charge is reached. After that point, the voltage curves begin to sharply plummet, and the smartphone can switch off automatically at any moment in order to protect the battery. The exact instant at which this disconnection occurs (and, consequently, the remaining lifetime) arbitrarily changes depending on several factors. This measured actual lifetime has also been contrasted (with satisfactory results) with the records obtained by the Diagnosis-System Information application when the same tests (in the same working conditions) are applied. Results indicate that the estimations of the battery lifetime offered by this application follow a linear time decay, at least until a depth of discharge of 95% is achieved. From these comparisons, we can conclude that the estimation of this “worst-case” battery autonomy is accurate.

To avoid the impact of other applications in the smartphones, the system is rebooted before the start of any test, while no additional application is installed or run on the smartphone (except for the diagnosis measuring routine). The computational costs of this measuring application were proven to be negligible, so it interfered neither with the battery lifetime nor with the normal development of the monitoring operation.

During all the experiments, the SIM (subscriber identity module) card is removed to avoid any consumption linked to the operation of the cellular communications. Thus, the particular conditions of the mobile network coverage do not affect the results either.

Regarding the Bluetooth communications, all the devices have been located within a radius of 1 m around the smartphone (the typical distance in a WPAN scenario). As the tests were conducted in an isolated building (a cottage), the absence of any other interfering Bluetooth connection is guaranteed. In any case, the technique of adaptive frequency hopping (AFH) is active in the BT interfaces. Similarly, the signal strength of all external Wi-Fi signals was measured to be under −85 dBm (normally below the operative reception threshold of most interfaces). Consequently, the possibility of interference is minimal, and the practical impact of other devices operating with the same radio technologies can be neglected.

The communication with the data network (Internet) is implemented via Wi-Fi. In particular, a Wi-Fi access point (located within a radius of 3 to 5 m in the line-of-sight of the smartphone) is specifically utilized for the experiment. The access point does not support any other background traffic.

The following subsections comment and detail the results of these three types of benchmarking tests.

### 4.1. Connectivity Capacity

These tests were intended to evaluate the actual connectivity capability of the smartphone. In all the cases, the WPAN operates in a fully configured mode, *i.e.*, with the whole set of sensors: the pulse oximeter, the ECG sensor, the Arduino-compass module and the blood pressure monitor. For comparison purposes, we employed a wide set of popular commercial Android smartphones and tablets from different vendors (see Table 1). For each device, the table specifies the employed versions of Android, as well as the Bluetooth controller firmware. It should be mentioned that for the HTC Desire smartphone, the official version (2.2) has been replaced by the unofficial and more updated 4.1.2 version.

The results indicate that the connection with the four Bluetooth sensors was properly established, except for the case of the Asus Transformer Tablet, which exhibited authentication problems with the Arduino module (this problem also appears if the connection is directly initiated from the operating system of the smartphone).

### 4.2. Benchmarking as Health Monitor

This subsection presents the results of the tests that were executed to characterize the consumption when the smartphone is acting as a health monitor exclusively (without executing any other operation). From the devices used in the connectivity experiments of the previous section, we selected as the test terminal the HTC Desire model (with Android OS version 4.1.2), as it presents more limited capabilities.

The profiling in the local mode for this device has been carried out in the scenarios indicated in Table 2 (TL, test for local mode). This table specifies for every test the profile and the mode in which the pulse-oximeter was configured. Additionally, we have conducted an exhaustive benchmarking of the remote operational mode of the application (*i.e.*, the mode where the smartphone acts as a gateway between the sensors and the central server). For this mode of operation, the performed tests were structured in the categories listed in Table 3 (TR, test for remote mode). For all the tests, the communication to the server was accomplished through a Wi-Fi connection.

It should be remarked that:

- In all cases, the data from each sensor are transmitted as byte arrays and are encapsulated in the body of independent HTTP POST requests.
- In the Table 3, the column labeled as “Transmission Mode” indicates the transmission mechanism that has been employed to retransmit the sensors' data to the central server. The performed tests consider the following transmission modes:
  – Continuous transmission (TR1, TR2 and TR3 tests): The available data in the smartphone are periodically sent to the central server irrespective of the number of connected devices. The transmission period is set to 1 s.
  – Data bursts of 10 s (TR4 and TR6 tests): All the data coming from a single sensor (the pulse-oximeter) are stored by the smartphone in a temporal buffer and re-transmitted to the server every 10 s. Data are inserted in the body of a HTTP POST request.
  – Asynchronous bursts of 10 s (TR5 and TR7 tests): In the smartphone, the data are saved in different buffers depending on the origin (*i.e.*, the sensor: pulse-oximeter or ECG). Every ten seconds, each buffer (which is managed by a different class) is emptied, and all the corresponding data are transmitted to the server. The information is again transported in the body of HTTP POST messages.
  – Synchronous bursts of 10 s (TR8 & TR9 tests): The data from the sensors are initially stored in different buffers, but then queued in a common queue every 5 s (double-buffering). The class responsible for the data transmission accesses this queue periodically (every 12 s). Then, the sensors' data are encapsulated in individual POST messages (depending on the source), but consecutively transmitted. Thus, the information from the whole set of sensors is received in a sequential way.
    Note that this method aims at improving the efficiency of the wireless transmissions. Firstly, storing the information received from each sensor (in a differentiated queue) permits aggregating and encapsulating more data from the same server in a single HTTP request. Thus, the method requires fewer requests than the case when they are retransmitted as soon as they are available. Secondly, the use of a unique timer for managing the shared queue permits consecutively transferring the HTTP messages containing data from all the sensors. Thus, the Wi-Fi transceiver can remain inactive for a longer period of time than in the previous mode where the transmissions of the data from the different sensors are asynchronously scheduled.
- We performed several tests (TR6, TR7 and TR9) to check the performance of the system when data compression is considered to reduce the transmitted traffic. In these cases, the utilized compression method was the the DEFLATE standard algorithm provided by the package “java.utile.zip.Deflater” [32].
- The classes that implement the transmission to the CCS do not run this task in the background (*i.e.*, they do not utilize the AsyncTask interface [33]).

The main results for the local and remote operational modes are summarized in Tables 4 and 5, respectively. In both tables, a specific column informs about the battery lifetime of the smartphone obtained for every scenario under test. Additionally, in the case of the remote monitoring, the table also indicates the achieved data rate (in bytes per second) between the smartphone and the CCS.

From the results of the tests performed for the different operation modes, we conclude that:

- In the local mode (without retransmissions) and if the pulse-oximeter is the only connected sensor:
  – Under the SPP profile and with the basic mode (M #8), the battery of the smartphone can operate more than two consecutive days (a total of 53.9 h) without requiring to be recharged.
  – Under the SPP profile and with the extended (verbose) mode (M #2), the battery lifetime was measured to be 36.8 h. This value clearly exceeds the lifetime (less than 12 h) of a former version of the prototype developed with Java and Python programming for a Symbian OS smartphone [34].
  – When comparing the results of the HDP profile with those achieved by SPP (under the basic configuration), the autonomy clearly decreases even when the same information (heart rate and oxygen saturation) is being transferred. In particular, the battery lifetime with SPP is 50% longer than that achieved with the HDP profile.
- In the local mode, but maintaining three Bluetooth connections with the three sensors, the pulse-oximeter, the ECG and the Arduino-compass module, results show that the smartphone can be operative for a period of up to 20 h.
- Under the remote mode, with a single sensor (pulse-oximeter, under the SPP profile), storing data for a period of 10 s before the transmission can increase the autonomy in 10 h, since the duration of 24.1 h estimated for the TR1 test is improved to 34.8 h in the TR4 test. Conversely, the compression of data executed in the TR6 test yields an expected battery lifetime of 31.8 h, reducing the gain with respect to the case of continuous sending without compression to a maximum of 7 h.
- We have to remark that, with a continuous transmission (TR1) and with bursts from a single sensor (TR4) the battery lifetime is 24.1 and 34.8 h, respectively. These values are much higher than those measured for a similar application developed for the Symbian OS platform with Java or Python languages [34]. In the best tests of the second scenario, the battery did not last longer than 10 h.
- In contrast with the case with just one sensor, when two sensing devices (the pulse-oximeter (under SPP) and the ECG) are simultaneously employed in the remote mode, the lifetime is not improved by queuing the data received from the sensor. We can appreciate this fact if we compare the battery duration obtained with asynchronous bursts (23.3 h, test TR5) with that measured when the sensors' data are retransmitted as soon as they are received by the smartphone (23.2 h, test TR3). Besides, the compression of the data stored every 10 s (test TR7) does not result in a significant increase (just one hour) of the battery duration. In any case, the compression allows one to reduce the transfer rate (568 bytes/s) with respect to the case where the uncompressed data are sent to the server (965 bytes/s, test TR5).
- The synchronization and sequencing of the transmissions of the data received from the two sensors (the pulse-oximeter (under SPP) and the ECG; test TR8) enable a slight increment of the battery lifetime with respect to the cases (tests TR3 and TR5) where the sending of the data in the queues assigned to the different sensors is not synchronized. However if this synchronization is combined with the data compression (test TR9), the autonomy of the smartphone is improved and increased up to three hours when compared to tests TR3 and TR5.
- The use of the GPS device has a great impact on the duration of the battery. Thus, when the location data (along with the pulse oximetry information) is also transmitted to the server, the battery lifetime is reduced to less than 12 h.

Finally, regarding the use of the CPU and the computational cost of the monitoring activities in the smartphone, it should be noted that in all cases, the application does not require more than 10% of the available processing capacity.

### 4.3. Compatibility with the Standard Functionality of the Smartphone

To evaluate the compatibility of the system with the normal operation of the smartphone, two scenarios have been envisaged: (i) reproduction of a music file; (ii) a phone-call. The results are described in the following paragraphs.

#### Compatibility with the reproduction of an audio file through the A2DP profile

We have used the phone as a music player connected to a Bluetooth headset that implements the A2DP (advanced audio distribution profile). This profile, which is aimed at supporting audio streaming applications, requires one to reserve bandwidth during the connection establishment to guarantee a certain QoS (Quality of Service) to the audio flows. The profile was chosen, as this strict demand of bandwidth resources can affect the coexistence with other devices and profiles operating in the same Bluetooth network (piconet). The goal was to evaluate the actual limitations of the smartphones when acting as masters of a piconet and keeping several simultaneous connections with heterogeneous devices and profiles: the headphones (A2DP) and the medical monitoring devices (SPP). From the results obtained in the tests (executed for both the local and remote operational modes), we conclude that:

- Local mode: All the Bluetooth connections of the fully configured WPAN (with the whole set of medical sensors) are kept operative. Just sporadic and punctual malfunctions and breaks are detected in the audio playback. With these operation conditions, the battery lifetime was estimated to be 19 h.
- Remote mode: Results indicate that the smartphone properly supports the coexistence of the operation of audio playing and its role as a gateway between the medical Bluetooth sensors and the central server. Thus, the audio sequence is reproduced without noticeable degradation, while the sensor data are received via Bluetooth and retransmitted via Wi-Fi without interruption. Under this remote mode, the estimated battery autonomy was about 17 h.

#### Compatibility with phone calls

The performed tests with the different models show that phone calls are not affected by the operation of the smartphone in the medical WPAN, both for the local and remote modes.

## 5. Conclusions

Smartphones are emerging as a low-cost and feasible technology for the rapid deployment of telecare applications. However, the actual potentials of smartphones in mHealth (mobile health) scenarios must be carefully analyzed. This paper has presented a thorough technical evaluation of a medical wireless personal area network (WPAN) based on Bluetooth communications and commercial Android smartphones. For this purpose, the paper proposes and describes a prototype of a telemonitoring system in which a set of wireless medical sensors are coordinated by a smartphone. In the proposed architecture, the smartphone typically acts as an information gateway, which alternatively behaves as a data Holter or gathers the information from the sensors via Bluetooth and retransmits them to a remote server through a Wi-Fi link.

The study has focused on benchmarking the smartphone that acts as the network coordinator node in the Bluetooth WPAN, as it is used for a completely different function than that for which it was conceived. The performed tests have evaluated different aspects, such as the connectivity capacity of the mobile device, the behavior of the smartphone as a health monitor and the compatibility of the WPAN with the standard functionality of the phone. Results indicate that factors, such as the selected Bluetooth profile, the inclusion of GPS devices in the WPAN or the use of data buffering, may heavily impact the battery lifetime. Conversely, other factors, such as the data compression, do not seem to introduce any significant improvement, with the exception of being combined with a double buffering technique. In any case, the measured autonomy of the Android smartphones (more than 30 h) is shown to be clearly higher than that obtained with a similar prototype developed on Symbian OS platforms.

On the other hand, the tests show that the use of the smartphone as a gateway in a WPAN may be compatible with the conventional functionality of the device (*i.e.*, audio file playback), even when Bluetooth and Wi-Fi communications are simultaneously active.

From the performed tests, we can conclude that present commercial mobile smart terminals are capable enough to combine their normal operation as phones or multimedia reproducers and simultaneously perform as medical monitors or gateways in an mHealth WPAN. In fact, in many of the successful performed tests, a basic single-core model (discontinued in 2011) was utilized. We can assume that the natural evolution of the smartphone technology will clearly reinforce this capability in the future.

Future studies will take into account the evolution of the wireless interfaces commonly provided by commercial smartphones. As soon as low-energy Bluetooth 4.0 will be available in both medical sensors and smartphones (only some incorporate BT 4.0, but it is not officially supported by Android), this performance evaluation should be repeated. In our testbed, the adoption of this new version of Bluetooth would require just the reprogramming of the communication module with the new sensor devices. In this new context, the utilized benchmark approach should not necessarily be modified.

In addition, some important aspects of a mHealth service, such as the usability analysis, are still open issues in the ambit of smartphone-based medical WPANs. Moreover, this work is being extended to investigate the feasibility of substituting the central server by a cloud-computing service. In this new scenario, the role of the smartphone as a gateway should be carefully characterized.

## Figures and Tables

**Table 1.** Android devices.

**Android Device**	**Android Version**	**Bluetooth Version**
Galaxy Nexus	4.2.1	4.0
Samsung Galaxy SIII	4.1.1	4.0
Samsung Galaxy SII	4.0.4	4.0
Samsung Galaxy Note	4.0.3	3.0
HTC One X	4.1.1	4.0
Asus Transformer	4.1.1	2.1
HTC Desire	4.1.2	2.1
HTC Sensation	4.0.3	2.1
Samsung Galaxy Note 10.1	4.1.2	4.0

**Table 2.** Test cases for the local (“Holter-like”) mode. SPP, serial port profile; HDP, health device profile.

**Test**	**Pulse-Oximeter Configuration**	**ECG**	**GPS**	**Compass**
TL1	SPP profile-Basic Mode			
TL2	SPP profile-Verbose Mode			
TL3	SPP profile-Verbose Mode			
TL4	SPP profile-Verbose Mode	**✓**		
TL5	SPP profile-Verbose Mode	**✓**		**✓**
TL6	HDP profile			

**Table 3.** Test cases for the remote mode.

**Test**	**Pulse-Oximeter**	**ECG**	**GPS**	**Transmission Mode**
TR1	Verbose Mode			Continuous
TR2	Verbose Mode		**✓**	Continuous
TR3	Verbose Mode	**✓**		Continuous
TR4	Verbose Mode			Bursts 10 s
TR5	Verbose Mode	**✓**		Asynchronous Bursts 10 s
TR6	Verbose Mode			Bursts 10 s; compression
TR7	Verbose Mode	**✓**		Asynchronous Bursts 10 s; compression
TR8	Verbose Mode	**✓**		Synchronous Bursts 10 s
TR9	Verbose Mode	**✓**		Synchronous Bursts 10 s; compression

**Table 4.** Test case results for the local mode.

**Test**	**CPU Application (%)**	**Battery Life (h)**	**Relative Gain (Referring to the Worst Case)**
TL1	4.4	53.9	4.45
TL2	4.6	36.8	3.04
TL3	6.1	12.1	1.00
TL4	5.0	29.8	2.46
TL5	5.2	20.9	1.73
TL6	6.7	35.3	2.92

**Table 5.** Test cases results for the remote mode.

**Test**	**CPU Application (%)**	**Transmission Rate (B/s)**	**Battery Life (h)**	**Relative Gain (Referring to the Worst Case)**
TR1	5.7	938.1	24.1	2.15
TR2	6.3	1,010.6	11.2	1.00
TR3	7.9	1,700.8	23.2	2.07
TR4	4.7	456.6	34.8	3.11
TR5	7.7	965.0	23.3	2.08
TR6	5.3	280.5	31.8	2.84
TR7	5.8	568.6	24.7	2.21
TR8	6.3	1,074.8	25.0	2.23
TR9	6.5	738.5	26.3	2.35
